# Excitotoxic stimulation activates distinct pathogenic and protective expression signatures in the hippocampus

**DOI:** 10.1111/jcmm.16864

**Published:** 2021-08-20

**Authors:** Ebru Caba, Marcus D. Sherman, Karen L. G. Farizatto, Britney Alcira, Hsin‐wei Wang, Charles Giardina, Dong‐Guk Shin, Conner I. Sandefur, Ben A. Bahr

**Affiliations:** ^1^ Vertex Pharmaceuticals Cambridge MA USA; ^2^ Department of Pharmaceutical Sciences and the Neurosciences Program University of Connecticut Storrs CT USA; ^3^ Biotechnology Research and Training Center University of North Carolina‐Pembroke Pembroke NC USA; ^4^ Department of Biology University of North Carolina‐Pembroke Pembroke NC USA; ^5^ Bioinformatics and Biocomputing Institute University of Connecticut Storrs CT USA; ^6^ Department of Computer Science and Engineering University of Connecticut Storrs CT USA; ^7^ Department of Molecular and Cell Biology University of Connecticut Storrs CT USA; ^8^ Department of Pharmacology and the Cystic Fibrosis and Pulmonary Diseases Research and Treatment Center University of North Carolina‐Chapel Hill Chapel Hill NC USA; ^9^ Sandefur Modeling Pittsboro NC USA; ^10^ Department of Chemistry and Physics University of North Carolina‐Pembroke Pembroke NC USA; ^11^ Present address: CureVac Inc. 250 Summer Street Boston MA USA

**Keywords:** compensatory signalling, excitotoxic response, gene array, neuroprotection, repair pathway, transcription factor

## Abstract

Excitotoxic events underlying ischaemic and traumatic brain injuries activate degenerative and protective pathways, particularly in the hippocampus. To understand opposing pathways that determine the brain's response to excitotoxicity, we used hippocampal explants, thereby eliminating systemic variables during a precise protocol of excitatory stimulation. N‐methyl‐d‐aspartate (NMDA) was applied for 20 min and total RNA isolated one and 24 h later for neurobiology‐specific microarrays. Distinct groups of genes exhibited early vs. delayed induction, with 63 genes exclusively reduced 24‐h post‐insult. Egr‐1 and NOR‐1 displayed biphasic transcriptional modulation: early induction followed by delayed suppression. Opposing events of NMDA‐induced genes linked to pathogenesis and cell survival constituted the early expression signature. Delayed degenerative indicators (up‐regulated pathogenic genes, down‐regulated pro‐survival genes) and opposing compensatory responses (down‐regulated pathogenic genes, up‐regulated pro‐survival genes) generated networks with temporal gene profiles mirroring coexpression network clustering. We then used the expression profiles to test whether NF‐κB, a potent transcription factor implicated in both degenerative and protective pathways, is involved in the opposing responses. The NF‐κB inhibitor MG‐132 indeed altered NMDA‐mediated transcriptional changes, revealing components of opposing expression signatures that converge on the single response element. Overall, this study identified counteracting avenues among the distinct responses to excitotoxicity, thereby suggesting multi‐target treatment strategies and implications for predictive medicine.

## 1 INTRODUCTION

Excitotoxicity, related to ischaemic stroke, traumatic brain injury and seizures, occurs through over‐activation of excitatory glutamate receptors. The glutamatergic receptors involved are those selectively activated by either N‐methyl‐D‐aspartate (NMDA) or α‐amino‐3‐hydroxy‐5‐methyl‐4‐isoxazolepropionate (AMPA), and their excessive activation causes neuropathology.^1^ Interestingly, glutamatergic activity can induce repair signalling. For instance, excitotoxic stimulation of NMDA receptors induces neuronal impairment and death, but in the developing brain these receptors play a role in neuronal survival.^2^ In addition, related signalling avenues have been suggested to promote cell survival in different in vitro and in vivo models.^3^, ^4^, ^5^ Thus, identifying competing genetic programmes is important to fully understand the influence of opposing pathways underlying the brain's response to injury.

The brain's response to an excitotoxic injury involves pathogenic and protective pathways, and clinical outcome is likely determined by the temporal relationships between such counteracting gene responses. Excitatory over‐activation, in particular, activates such opposing pathways, with excitotoxicity causing transcriptional induction of both cell death‐ and cell survival‐related genes.^6^, ^7^ Examples of differential gene expression during cerebral ischaemia and models of excitotoxic events were demonstrated by measuring brain RNA expression profiles.^7^, ^8^, ^9^, ^10^ Notably, temporal expression changes were identified in the transient middle cerebral artery occlusion (MCAO) stroke model by employing neurobiology‐focussed arrays.^7^ The MCAO study provides strong evidence of distinct acute versus delayed gene expression profiles comprised of diverse functional categories.

The present study investigates the excitotoxic injury response in the hippocampus using the cultured hippocampal slice, a physiologically relevant three‐dimensional tissue model with native pathogenic responsiveness with regard to neurodegenerative insults.^3^, ^4^, ^11^, ^12^, ^13^ Different excitotoxic insults in the explants indeed resulted in the expected delayed neuronal damage along with associated compensatory signals found involved in governing cellular damage.^3^, ^4^, ^6^ The hippocampus is particularly important to study as it is a higher brain centre involved in memory encoding and behavioural responses, as well as being distinctly vulnerable to excitotoxicity. Here, excitotoxicity was induced through activation of NMDA receptors and the associated transcriptional responses were assessed one hour (early) and 24 h (delayed) later with Affymetrix focussed arrays containing 1300 neurobiologically relevant sequences. The utilization of categorical filtering was an additional informatics step to identify transcriptional regulation events with high propensity of being involved in cellular degeneration versus cellular protection. Networks of co‐expressed neurobiology‐relevant genes were computationally generated and found to be in congruence with the temporal patterns observed using the experimental hippocampal slice cultures. Additionally, Gene Ontology annotations demonstrated unique functional networks involving the early and delayed expression profiles.

## 2 METHODS

### 2.1 Organotypic hippocampal slice cultures

All of the studies were carried out in strict accordance with Animal Welfare Act and other Federal statutes and regulations related to animals and their use. The procedures adhered to recommendations from the Guide for the Care and Use of Laboratory Animals of the National Institutes of Health. Animal use and analyses were conducted in accordance with approved protocols of the Animal Care and Use Committees of the University of Connecticut and the University of North Carolina–Pembroke. Sprague Dawley rat litters (Charles River Laboratories; Wilmington, Massachusetts) were housed following guidelines from the National Institutes of Health and approved animal protocols. Interface hippocampal slice cultures were prepared from animals at 12 days postnatal. Transverse slices from cooled hippocampi (400 µm) were quickly prepared, maintained on Millicell‐CM inserts (Millipore Corporation; Bedford, Massachusetts) and periodically supplied with media composed of 50% basal medium Eagle, 25% Earle's balanced salts, 25% horse serum and defined supplements.^12^, ^13^ Slices were maintained at 37°C, and media changes occurred every 2–3 days. Slices were allowed to mature for 16–20 days in culture before being used in experiments. For immunostaining, subsets of cultured slices were quickly harvested for homogenate preparation and immunoblotting with specific antibodies, or they were cold fixed in 4% paraformaldehyde for 4 h, placed in 20% sucrose overnight and sectioned at 20‐µm thickness for immunohistochemistry protocols.

### 2.2 Induction of excitotoxicity

The hippocampal slice model of excitotoxicity involves the excitotoxic insult achieved by infusing the long‐term cultured slices with serum‐free media containing 200 μM NMDA (Tocris; Ellisville, Missouri), a sufficient concentration to ensure rapid activation of NMDA receptors throughout the three‐dimensional tissue cultures. For a consistent 20‐min insult period, the excitatory activation was quickly stopped using multiple media washes containing 40 μM each of MK801 and CNQX (Tocris), two selective antagonists to quench any further activity of NMDA and AMPA receptors, respectively. In separate experiments, cultured hippocampal slices were treated with 60 μM MG‐132, a proteasome inhibitor, for 60 min before as well as during and after the NMDA infusion and quenching steps. The slice cultures were then placed in fresh media for the post‐insult period of 1–24 h. Start times were adjusted such that all treated and control slices were collected on the same day, harvesting with a gentle brush to remove them from the culture inserts.

### 2.3 Gene array preparation and hybridization

Groups of 40 hippocampal slices each were harvested at 1 or 24 h after NMDA exposure for microarray analyses (3 groups per experimental condition). Total RNA was extracted in TRIzol^®^ (Invitrogen, Carlsbad, California), and the concentration was determined by spectrophotometric analysis. Equal amounts of total RNA (15 μg) from the samples were used for cDNA library construction using SuperScript Double‐Stranded cDNA synthesis kit (Invitrogen) and the reverse transcription T7‐(dT)_24_ primer (IDT, Coralville, IA). Each cDNA preparation was extracted with phenol/chloroform, ethanol precipitated and then used as a template for in vitro transcription to synthesize biotin‐labelled cRNA with the ENZO BioArray High Yield RNA transcript Labeling Kit (Affymetrix, Santa Clara, California). After determining concentrations with OD_260_/OD_280_ measures, the cRNA preparations were fragmented by heating for 35 min. The fragmented cRNA (15 μg) from each sample was hybridized to U34 Rat Neurobiology GeneChip arrays (Affymetrix) for 16 h, and the hybridization, staining, scan and analysis were conducted as recommended by Affymetrix protocols.

To verify the gene array results, real‐time quantitative reverse transcription‐polymerase chain reaction (qRT‐PCR) was carried out on selected genes in NMDA‐treated and control samples. Extracted total RNA was assessed for concentration as determined from optical density measurement at 260 and 280 nm. Routine two‐step qRT‐PCR analyses were then conducted in duplicate with an Applied Biosystems detection system, utilizing TaqMan assays specific to the rodent genes being assessed.

A subset of hippocampal slices harvested 0.5‐ to 40‐h post‐insult was also subjected to nuclear extraction after rapid homogenization in ice‐cold nuclear extract buffer. For measures of MG‐132‐sensitive NF‐κB activation, equal aliquots of the nuclear extracts (7.5 μg protein) were subjected to the electrophoretic mobility shift assay (EMSA) described previously,^6^ using a labelled NF‐κB consensus oligonucleotide probe.

### 2.4 Annotation and analysis of gene array data

Over 1300 sequences relevant to neurobiology research are included in the Affymetrix microarray design. Statistically significant alterations to RNA transcript levels relative to control slices were determined using the Affymetrix Genechip software MAS 5.0. Greater than 50% induction or repression in expression was applied as a filtering threshold among those genes identified by the software to have a statistically significant change. For each treatment, three gene chips were used and compared with corresponding sham samples. Differentially expressed gene transcripts were sorted through complete linkage clustering using a Euclidean distance measurement transformed into heat maps using R.^14^ Filtered gene sets were also subjected to gene‐by‐gene literature searches and divided into functional classes. Genes linked to predominately pro‐survival pathways versus predominately pathogenic pathways were categorized to assemble gene profiles of potential opposing pathway responses. Co‐expressed genes were determined using the GeneMania database.^15^ Gene ontological (GO) terms were determined using the DAVID database^16^ and the PANTHER database.^17^ All networks were constructed using Cytoscape (created by Institute for Systems Biology; provided by U.S. National Institute of General Medical Sciences).

A large body of literature guided categorical filtering, with the following genes being listed with exemplary references for their consensus linkage to cell survival: HO‐1,^18^ Egr‐1,^19^ IL‐6,^20^ SOCS‐3,^21^ c‐fos,^22^ JunB,^23^ cyclin L,^24^ HES‐1,^25^ HSP27,^26^ HSP10,^27^ JAK2,^28^ IGF II,^29^ IGF II BP3,^29^ BDNF,^30^ neuronatin α,^31^ neuro‐D4,^32^ trkB,^33^ PKCβ,^34^ PKCγ^35^ and ILK.^36^ The list of genes linked to pathogenesis includes the following: cJun,^37^ IL‐1β,^38^ NGFI‐B,^39^ caspase 1 (previously known as interleukin‐1β‐converting enzyme or ICE),^40^ NOR‐1,^41^ fra‐1,^42^ ICAM‐1,^43^ MIP‐1α,^44^ C/EBP,^45^ IRF‐1,^46^ CREM,^47^ nNOS,^48^ TNF‐α,^38^ CX3CL1^49^ and calpain.^1^

## 3 RESULTS

**FIGURE 1:**
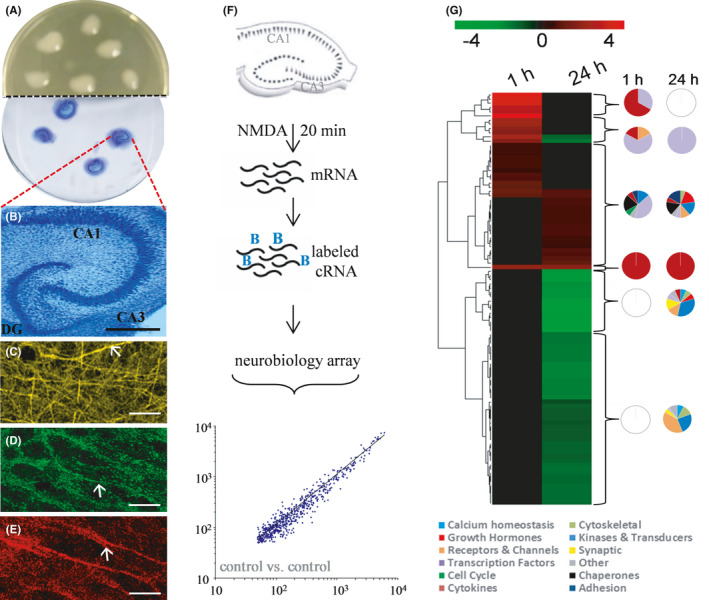
NMDA‐mediated excitotoxicity in stable hippocampal explants to measure transcriptional responses. Organotypic cultures of rat hippocampal slices were maintained on the Biopore membrane of Millicell inserts (A). The Nissl‐stained slice cultures have preserved organization of the major neuronal subfields (B), and the neurons were stained for βIII‐tubulin (C) and found to be densely populated with synapses positive for the markers synaptophysin (D) and GluR‐1 (E). Size bars: B, 280 μm; C‐E, 24 μm. The excitotoxic insult protocol and gene expression profiling consisted of a 20‐min infusion of 200 μM NMDA, rapid antagonist quenching of the cultures, followed by RNA isolation and synthesis of biotinylated cRNA (F). Using chip hybridization with Affymetrix Neurobiology arrays, expressed genes were found to have a close linear distribution of hybridization signal intensities between like treatment groups (*R* ^2^ = 0.959 for the lower scatter plot). For explants harvested 1 h and 24 h after the NMDA‐induced excitotoxic stimulation and compared with vehicle treatment, expression profiles were visualized using a heat map for the most significant gene clusters (G). Differential expression between the 1‐ and 24‐h post‐insult groups was found, with green, black and red indicating low, intermediate and high gene expression, respectively. Dendrograms were formulated through complete linkage clustering using a Euclidean distance measurement. Pie charts for each cluster depict percentages of those genes in the listed functional categories

For the study of transcriptional events elicited specifically in the hippocampus, we prepared rat hippocampal slices and maintained them in culture on Millipore inserts (Figure 1A). The explants displayed long‐term maintenance of the major neuronal subfields of the hippocampus (Figure 1B) and their dense processes populated with evident staining of synaptic terminals (Figure 1C–E). Former studies reported that the slice cultures exhibit connectivity, plasticity and pathogenic responsiveness as found in vivo.^11^, ^13^, ^50^, ^51^, ^52^ Here, we used an NMDA infusion period that led to pathogenic changes as previously described.^6^, ^51^ The protocol for the excitotoxic stimulation and subsequent measuring of gene expression profiles is shown in Figure 1F, in which the explants were infused with NMDA for 20 min followed by rapid washout in the presence of glutamatergic antagonists for precise insult duration due to quenching any further excitatory activation.

To test for early versus delayed expression profiles after the defined excitotoxic insult, RNA was isolated from groups of slice cultures, cDNA was generated and used to synthesize biotinylated cRNA, which was followed by the chip hybridization protocol employing Affymetrix Rat Neurobiology U34 arrays. Across the approximately 1300 neurobiologically relevant sequences in the arrays, the scatter plot in Figure 1F indicates a close correlation of hybridization signal intensities between non‐treated control samples. The linear distribution of expression intensities confirms reproducible measures of transcription levels.

Compared with the expression levels measured in vehicle‐treated control slices, the NMDA insult led to 106 differentially expressed genes across the two post‐insult times. The small set from the 1300 neurobiologically relevant genes met the criteria of at least a 50% increase or decrease in expression and represented a wide range of functionality. In Table 1, functional information about the 106 genes was used to classify them under several broad titles, consisting of cell cycle proteins, cytokines and inflammatory agents, transcription factors, chaperons and heat shock proteins, receptors and channel proteins, synaptic proteins, kinases and signal transduction proteins, growth hormones, transporters and neurotransmitters, adhesion molecules, and calcium homeostasis. Clones that could not be placed under any of the headings were designated as other. The differentially expressed genes evident 1‐h vs. 24‐h post‐insult produced distinct heat maps (Figure 1G). The distinctions between the early and delayed transcriptional regulation events were further distinguished with dendrograms formulated through linkage clustering. To identify possible functional differences in the differentially expressed gene clusters, counts of functional categories in each cluster were completed and depicted as pie charts in Figure 1G. The pie charts express the per cent of regulated genes in each of the functional categories. The early transcriptional regulation events are distinct in that they are clustered in the cytokine and transcription factor gene groups, whereas the delayed events were mostly found outside those two classifications. The largest up‐regulation events included 11‐ to 50‐fold increases in four cytokines at 1‐h post‐insult, as well as early 13‐ to 24‐fold changes to two members of the transcription factor group (NGFI‐B and fos‐related antigen Fra‐1). The largest delayed increase in expression was a 4.9‐fold change to the chemokine monocyte chemoattractant protein (MCP).

**TABLE 1:** Functional classification of genes whose expressions are altered following excitotoxicity

Gene name	GenBank No.	LocusLink ID	Unigene Accession Number	Post‐insult time	
				1 h	24 h
Cell cycle proteins					
Cyclin L	AF030091	114121	12962	**2.56**	
Cytokines					
TNF‐α	L00981	25008	9820	**11.40**	
Interleukin 1‐β (IL1β)	M98820	24494	9869	**21.16**	
Interleukin 6 (IL−6)	M26744	24498	9873	**49.66**	
Caspase 1	S79676	25166	37508		**2.03**
Macrophage inflammatory protein−1α	U22414	25542	10139	**26.69**	
Macrophage inflammatory protein−2 precursor	U45965	114105	10230	**7.73**	
Monocyte chemotactic protein (MCP)	X17053	24770	4772	**5.81**	**4.92**
Chemokine (C‐X3‐C motif) ligand 1	AA800602	89808	107266		**0.13**
cAMP phosphodiesterase (PDE4)	M25350	24626	2485	**1.61**	
Transcription factors					
c‐fos	X06769	314322	103750	**4.62**	
c‐jun oncogene for transcription factor AP−1	X17163	24516	44320	**1.67**	
pJunB	X54686	24517	15806	**2.86**	
Egr−1	M18416	24330	9096	**8.11**	**0.42**
NGFI‐B	U17254	79240	10000	**12.57**	
Transcriptional repressor CREM	S66024	25620	10251	**1.74**	
C/EBP	X60769	24253	6479	**2.98**	**2.08**
Suppressor of cytokine signalling−3 (SOCS−3)	AF075383	89829	29984	**6.76**	
Interferon regulatory factor 1 (IRF−1)	M34253	24508	6396	**1.65**	
Transcription factor HES−1	D13417	29577	19727	**2.07**	
Fos‐related antigen (Fra−1)	M19651	25445	11306	**23.80**	
Brain finger protein (BFP) zinc finger protein 179	AF054586	24916	7544		**0.14**
Neuro‐D4	X66022	50545	42906		**0.48**
Nr4a3 (NOR)	AI176710		62694	**4.43**	**0.30**
NF‐kB (p105)	L26267	81736	2411		**1.66**
Chaperones and heat shock proteins					
Haem oxygenase (HSP32)	J02722	24451	3160	**2.66**	**2.23**
Heat‐shock 27 kDa protein	AI176658	24471	3841		**2.60**
Heat shock 10 kD protein 1 (chaperonin 10)	AI170613	25462	1540		**1.75**
Receptors & channels					
GluR−1	M36418	50592	29971		**0.27**
GluR−2	M38061	29627	11364		**0.27**
GluR‐K3	X54656	29628	74049		**0.06**
Homer, neuronal immediate early gene, 1	AF030088	29546	37500	**5.45**	
Neuronal activity‐regulated pentraxin (Narp)	S82649	25487			**0.46**
GABA‐B receptor gb2	AF058795	83633	30039		**0.28**
GABA‐A receptor α−1	L08490	29705	105630		**0.46**
Peripheral‐type benzodiazepine receptor (PKBS)	J05122	24230	1820		**2.02**
Degenerin channel MDEG	U53211	25364	37523		**0.26**
Sodium channel I	M22253	81574	32079		**0.40**
Sodium channel II	M22254	24766	10136		**0.25**
K+ channel Kv4.2	M59980	65180	10754		**0.18**
Calcium channel α−1 subunit	U14005				**0.48**
Pore‐forming calcium channel α−1B	AF055477	257648	85880		**0.41**
Potassium‐dependent sodium‐calcium exchanger (NCKX2)	AF021923	84550	74242		**0.29**
ATPase, Na+K+ transporting, β polypeptide 3	AA943304		5041		**1.71**
Potassium channel protein (RHK1)	M32867	25469	9884		**0.37**
Dihydropyridine‐sensitive L‐type calcium channel α−2 subunit (CCHL2A)	M86621	25399	11276		**0.26**
Neural receptor protein‐tyrosine kinase (trkB)	M55291	25054	11246		**0.48**
IP−3 receptor	J05510	25262	3841		**0.36**
Other synaptic proteins					
SNAP−25A	AB003991				**0.20**
Synaptophysin	X06655		11067		**0.27**
Synapsin 2	rc_AI145494		506		**0.11**
SV2 related protein (SVOP)	AF060173		30057		**0.29**
Adhesion molecules					
ICAM−1	D00913	25464	12	**1.59**	
Neurocan	M97161	58982	10177		**1.80**
C‐CAM4	U23056	287009	92160		**1.93**
Integrin αM	U59801	25021			**1.70**
Growth hormones					
VGF	M74223	29461	9704		**1.85**
IGFII gene for insulin‐like growth factor II	X17012	24483	964		**1.55**
Insulin‐like growth factor‐binding protein (IGF‐BP3)	M31837	24484	26369		**1.59**
BDNF	AI030286	24225	11266		**0.09**
Kinases and transduction molecules					
Carboxyl‐terminal PDZ ligand of nNOS	AF037071	192363	9903		**0.17**
MAP‐kinase phosphatase (cpg21)	AF013144	171109	10877	**2.00**	
Ca^2+^/calmodulin‐dependent protein kinase I β	AB004267	29660	11178		**0.11**
Ca^2+^/calmodulin‐dependent protein kinase II α	M16960	25400	98652		**0.25**
Ca^2+^/calmodulin‐dependent protein kinase II β	M16112	24245	9743		**0.05**
Integrin‐linked kinase	AI102079	170922	95042		**0.47**
Protein‐tyrosine kinase (JAK2)	U13396	24514	18909		**1.66**
Inositol (1,4,5) trisphosphate 3‐kinase (IP3K)	X56917	81677	9877		**0.05**
PKC gamma	M13707	24681	9747		**0.15**
PKC β	K03486	25023	91118		**0.38**
Alternatively spliced GTP‐binding protein α subunit (stimulatory) (GS‐α)	L10326	24896	31		**0.50**
Plasma membrane calcium ATPase‐isoform 1	L04739				**0.43**
Calpain II 80 kDa	L09120	29154	6822		**1.88**
Calcineurin A α	D90035	24674	6866		**0.38**
Ca^2+^ ATPase‐isoform 2	J03754	24215	90982		**0.30**
Calcium‐dependent tyrosine kinase 2 β	AF063890	50646	11025		**0.42**
Phospholipase C−1	M20636	24680	101292		**0.31**
Ras‐related rab3	X06889	25531	44409		**0.28**
c‐kit receptor tyrosine kinase	D12524	64030	54004		**0.37**
Bax	S76511	24887	10668		**1.50**
Cytoskeletal proteins					
Neurofilament, heavy polypeptide	AA818677	24587	1429		**0.36**
Neurofilament protein middle (NF‐M)	Z12152	24588	10971		**0.31**
Smallest neurofilament protein (NF‐L)	AF031880	83613	18658		**0.11**
Microtubule‐associated protein 2	X53455				**0.46**
Solute carrier family 1, member 3	AI101255	29483	34134		**0.36**
Sodium‐dependent neurotransmitter transporter	S56141				**0.43**
Class I β‐tubulin	AI229707	29214	2458		**2.21**
Calcium homeostasis proteins					
Neuron‐specific protein PEP−19	M24852	25510	9736		**0.26**
Neurogranin (RC3)	L09119	64356	11236		**0.10**
Hippocalcin	D12573	29177	11019		**0.26**
Neural visinin‐like Ca2+‐binding protein type 2 (NVP−2)	D13125	50872	34529		**0.34**
Other					
Synuclein SYN1	S73007	29219	1827		**0.19**
β‐synuclein	D17764	113893	20352		**0.46**
Tissue‐type plasminogen activator (tPA)	M23697	25692	1002	**1.59**	
Neuronatin α	U08290	94270	5785		**0.36**
Activity and neurotransmitter‐induced early gene 1 (ania−1)	AF030086			**2.78**	
Activity and neurotransmitter‐induced early gene 4 (ania−4)	AF030089	59104	40517		**1.80**
Cholecystokinin (CCK)	X01032	25298	9781		**0.35**
EST	AI228113				**0.25**

Note

In each case, the fold change is from comparisons between treated versus control tissue samples (*n* = 3 chips per group). The criteria for this table are discussed in the Materials and Methods section and include an average of a twofold change or greater in the treated to control comparison using the Affymetrix Genechip software MAS 5.0.

Abbreviations: ANIA, activity and neurotransmitter‐induced early gene; CREM, cAMP responsive element modulator; Egr‐1, early response factor 1; fra‐1, fos‐related antigen; HES‐1, hairy and enhancer of split 1; HO‐1, haem oxygenase‐1; HSP, heat shock protein; ICAM, intercellular adhesion molecule; IGF, insulin‐like growth factor; IL, interleukin; IP‐3, inositol triphosphate; IRF‐1, interferon regulatory factor 1; MAPK, mitogen‐activated protein kinase; MCP, monocyte chemoattractant protein 1; MIP, macrophage inflammatory protein; NF‐κB, nuclear factor‐κB; NGFI, nerve growth factor induced; NOR‐1, neuron‐derived orphan receptor; PDE4, cAMP phosphodiesterase; PTBR, peripheral‐type benzodiazepine receptor; TNF, tumour necrosis factor; tPA, tissue plasminogen activator.

**FIGURE 2:**
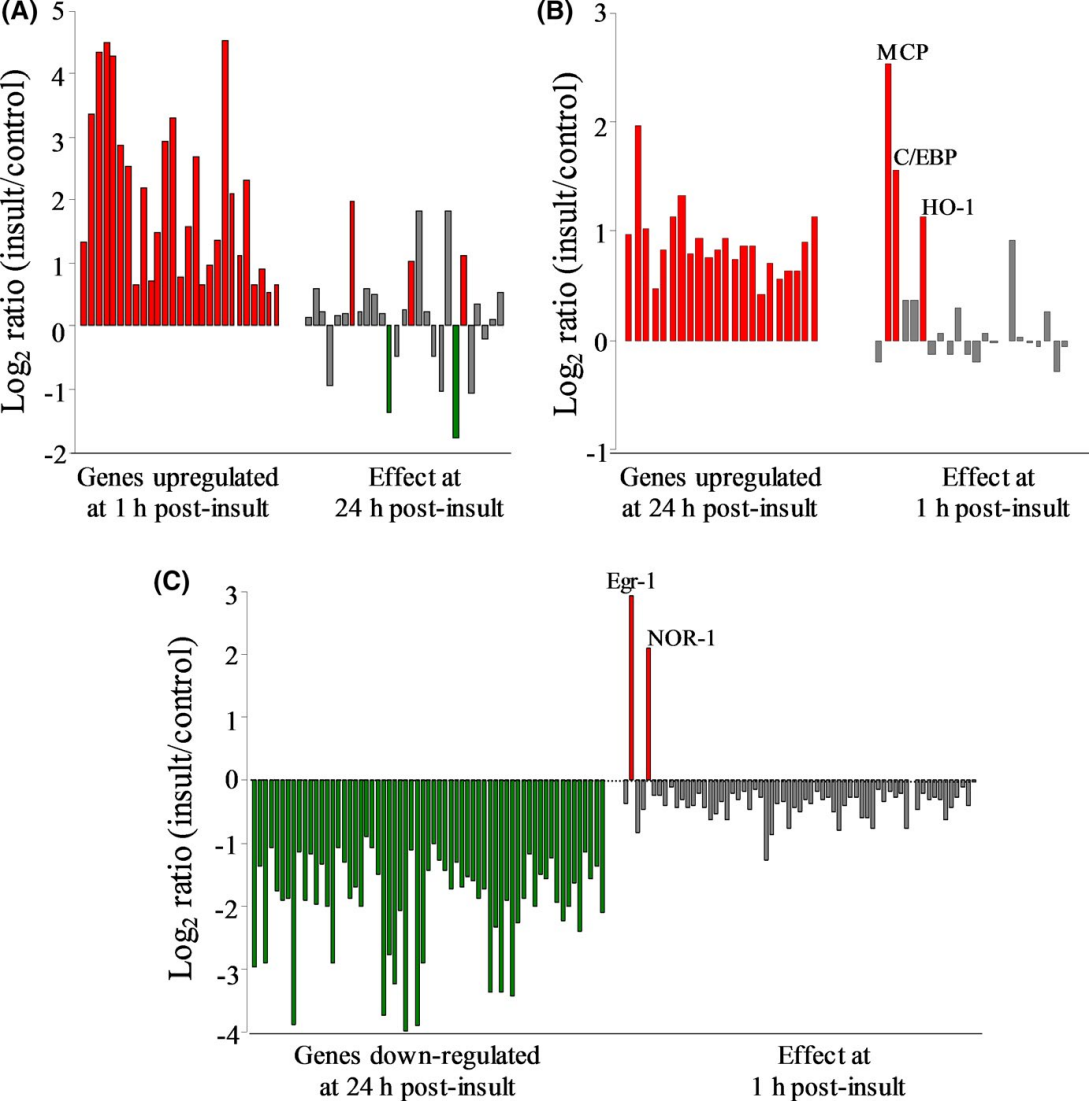
Cellular responses to excitotoxicity are defined by distinct gene expression profiles found early vs. delayed after the insult. RNA from control and NMDA exposed hippocampal slices was subjected to microarray analysis using Affymetrix GeneChips. (A) Genes that are up‐regulated early and their 24‐h counterparts. Genes that are (B) up‐regulated or (C) down‐regulated late (24 h) after the insult and their respective early counterparts. Note that green identifies those genes that were up‐regulated above 50% with a significant increase call. Red bars represent those genes that were down‐regulated 50% with a significant decrease call, while those genes that did not change or fit the 50% criteria, are shown as grey bars. Only those genes that were consistently altered in three independent repetitions are listed

Further profiles were constructed in order to test for distinct patterns of differentially expressed genes in response to the excitotoxic insult, as well as for those specific genes exhibiting unique transcriptional changes. Up‐regulation events at 1‐h post‐insult consisted of a distinct set of 26 transcripts (Figure 2A, red bars on left), and among these, only five exhibited statistically significant changes in expression 24 h after the NMDA exposure (right bars). Note that using the same order of genes listed for the 1‐h data, the 24‐h post‐insult responses consist mostly of grey bars determined to be outside statistical significance, along with two biphasic genes exhibiting delayed down‐regulation (green bars) and three genes with continued up‐regulation (red bars). The latter three exhibiting early and delayed up‐regulation were MCP, up‐regulated 5.81 ± 0.36‐ and 4.92 ± 2.46‐fold (mean ± SEM), the transcription factor C/EBP up‐regulated 2.98 ± 0.25‐ and 2.08 ± 0.23‐fold and haem oxygenase‐1 (HO‐1 or HSP32) up‐regulated 2.66 ± 1.16‐ and 2.23 ± 0.30‐fold, respectively.

At 24‐h post‐insult, a new pattern of 20 up‐regulated genes was found (Figure 2B), and this delayed pattern shares only MCP, C/EBP and HO‐1 with the profile of early up‐regulated genes. The remaining 17 genes in the delayed pattern were found to be statistically unchanged with regard to their early expression alterations. Thus, two distinct groups of genes with very little overlap exhibit increased expression levels 1 h vs. 24 h after the excitotoxic insult. The 24‐h post‐insult time was further distinctive by having 63 genes down‐regulated exclusively in a delayed manner (Figure 2C). The behaviour of these 63 genes at the early time found that two of the genes, early response factor 1 (Egr‐1) and the neuron‐derived orphan receptor‐1 (NOR‐1)—both transcription factors—displayed biphasic responses exhibited by early induction followed by delayed suppression. Egr‐1 displayed dramatic induction 1 h after the excitotoxic insult (eightfold), but that regulated level of expression was suppressed 95% in hippocampal slices harvested 24 h after NMDA treatment, reaching a level 58% below the baseline of vehicle‐treated slice cultures (0.42 ± 0.12 of control expression). NOR‐1’s early induction in the excitotoxic slice model was 4.42 ± 0.82‐fold after 1 h, followed by a 93% reduction in expression by 24 h (0.30 ± 0.05 of control). To verify these unique responses, qRT‐PCR analyses were conducted resulting in confirmation of biphasic transcriptional responses by Egr‐1 (early vs. delayed Log2 of 2.0 vs. −1.3) and NOR‐1 (1.9 vs. −0.82).

Interestingly, it should be pointed out that the excitotoxic hippocampal slice model was without any gene expression changes that met the minimal criteria of ≥50% decline at 1‐h post‐insult. Among the delayed down‐regulation events, 9 of the 63 genes were markedly reduced in expression by 89%–95%. The nine genes represent mostly synaptic proteins or proteins linked to synaptic mechanisms. Among the synaptic proteins, GluR‐K3 expression was reduced by 94% and synapsin 2 was reduced by 89% (see Table 1). SNAP 25B expression was reduced by 90% at 24‐h post‐insult and by 49% at the 1‐h time point, the early regulation being just shy of the filtering criteria. It should be noted that the related protein SNAP‐25A exhibited a delayed reduction in expression of 80%. In addition, Ca^2+^/calmodulin‐dependent protein kinase IIβ (CaMKIIβ) was reduced by 95%, neurogranin (RC3) by 90% and brain‐derived neurotrophic factor (BDNF) by 91% at 24‐h post‐insult.

**FIGURE 3:**
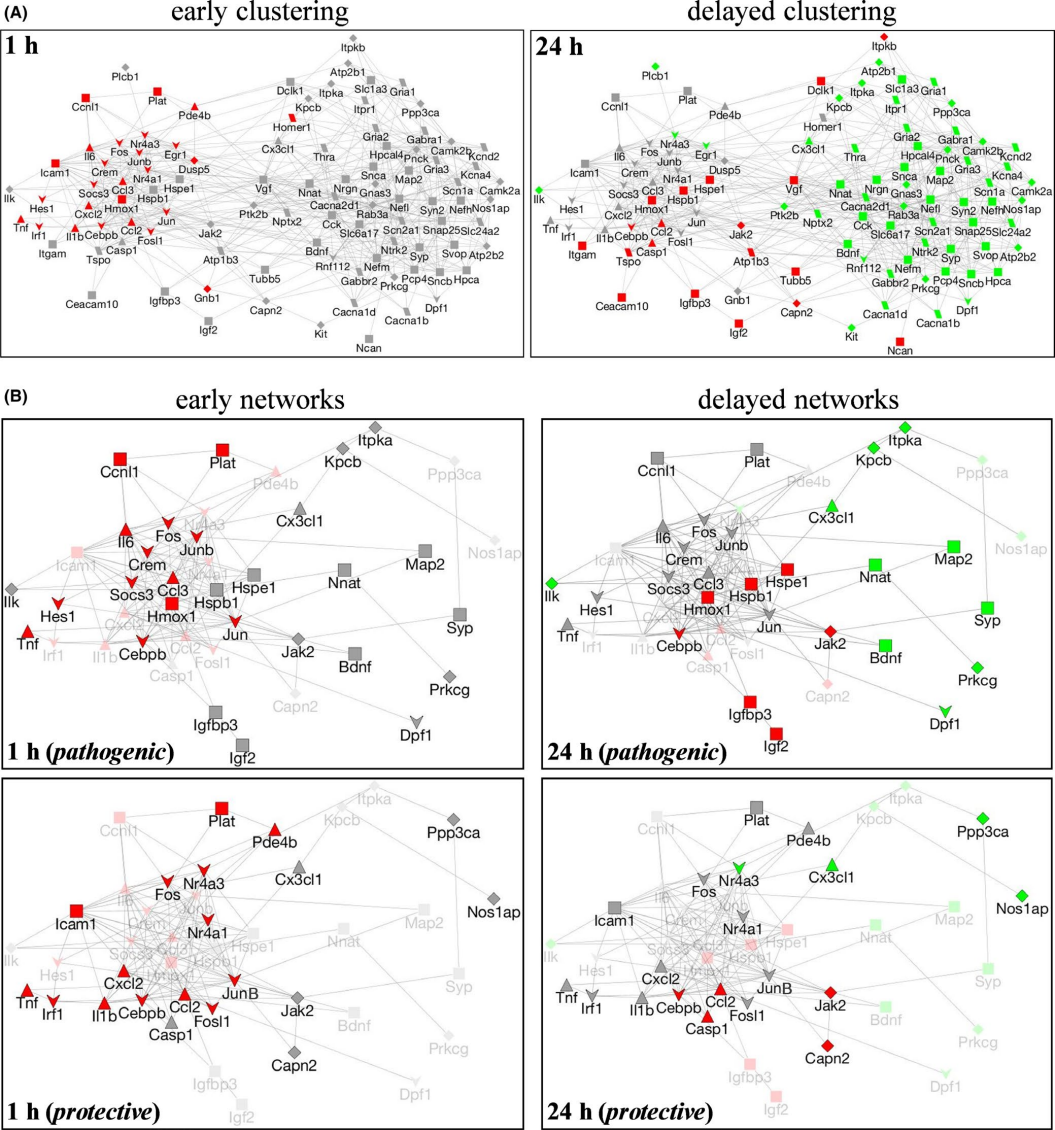
Gene networks associated with NMDA‐mediated excitotoxicity. Differentially expressed gene networks at 1‐h (left) and 24‐h post‐insult (right) mirror coexpression network clustering (A). Green nodes represent underexpressed genes, red denotes overexpressed genes, and grey denotes genes that have no measured change relative to control. Edges denote co‐expressed genes found via GeneMania. Node shapes denote the following categories: transcription factors (downward arrow), cytokines (triangle), receptors and channels (parallelogram), kinases and transduction (diamond), and all others (squares). Next, collecting genes by pathogenic and protective classification demonstrated that early and delayed pathogenic and delayed protective networks also resemble coexpression networks (B). As in panel A, green nodes represent underexpressed genes, red denotes overexpressed genes, and grey denotes no significant change relative to control explants

To identify networks of differentially expressed genes involved in early versus delayed responses, a gene coexpression network was generated using GeneMania. To test for differentially expressed gene networks involved in the early vs. delayed response to excitotoxicity, we created a single‐gene coexpression network using GeneMania,^15^ where each of the 103 differentially expressed genes is represented by a node. Edges denote evidence of gene coexpression found in previous studies in NCBI Gene Expression Omnibus via GeneMania. In this way, we informed our neuro‐specific gene expression network with prior gene expression studies but the network was not biased by cell or tissue type. As shown in Figure 3A, the generated network clustered into two distinct sets based on the gene coexpression data from the two post‐insult assessments. We then overlaid our early and late gene expression data onto our coexpression network. Interestingly, we found that our temporal gene profiles mirrored the coexpression network clustering, suggesting our insult‐induced neuro‐specific temporal gene expression pattern may be part of a more basic stress response pattern, which has been observed across a variety of gene expression studies. Distinct functional networks associated with the temporal profiles were also evident when mapping the genes to biological function using Gene Ontology databases. For example, as compared to early changes in biological function, delayed changes included cytoskeleton organization and neurotransmitter and ion transport, suggesting repair and energy‐dependent processes were taking place.

Both pathogenic and repair responses have been linked to excitotoxicity in the brain; therefore, categorical filtering was applied accordingly to the 106 differentially expressed genes. Over 40 of the 106 NMDA‐induced genes are commonly categorized as either primarily involved in pathology or primarily in cellular repair/survival. By assessing such categorical distinction across genes influenced at the two post‐insult times, discrete patterns of degenerative vs. repair events were identified (Table 2). The early degenerative signalling consists of 15 up‐regulated pathogenic genes, 9 of them being increased 3‐ to 27‐fold 1 h after the NMDA insult (MCP, C/EBP, NOR‐1, MIP‐1α, Fra‐1, IL‐1β, NGFI‐B, TNF‐α and MIP‐2) while CREM, cJun, IRF‐I, PDE4, ICAM‐1 and tPA were up‐regulated to a smaller degree (1.58‐ to 1.74‐fold). Of these 15 genes, only MCP and C/EBP exhibited enhancement of expression at the 24‐h post‐insult time as well, being among a small group of up‐regulated pathogenic genes that also included caspase 1, calpain II and bax. Also illustrated in Table 2, early degenerative changes appear to consist only of increased pathogenic genes, whereas delayed degenerative changes included increased pathogenic genes as well as down‐regulated expression of a putative set of survival genes negatively influenced by the excitotoxic stimulation (Egr‐1, neuro‐D4, trkB, PKCβ, neuronatin α, PKCγ, BDNF, IP3K and ILK). The qRT‐PCR analyses confirmed many transcriptional responses identified in the microarray data including the early, marked induction of NOR‐1, IL‐1β and Egr‐1, and the delayed down‐regulation of trkB and neuronatin α using specific primers.

**TABLE 2:** Categorical filtering identifies distinct patterns of degenerative and repair signalling

	Early versus delayed degenerative changes			
	1 h		24 h	
Increased pathogenic genes:	**MCP**	**5.81 ± 0.36**	**MCP**	**4.92 ± 2.46**
	**C/EBP**	**2.98 ± 0.25**	**C/EBP**	**2.08 ± 0.23**
	*NOR‐1	4.42 ± 0.82	caspase 1	2.03 ± 0.40
	MIP‐1α	26.7 ± 13.3	calpain II	1.88 ± 0.15
	fra‐1	23.8 ± 4.10	bax	1.50 ± 0.06
	IL‐1β	21.1 ± 4.65		
	NGFI‐B	12.5 ± 1.36		
	TNF‐α	11.4 ± 3.10		
	MIP‐2	7.72 ± 1.88		
	CREM	1.74 ± 0.07		
	cJun	1.67 ± 0.14		
	IRF‐I	1.65 ± 0.31		
	PDE4	1.61 ± 0.20		
	ICAM‐1	1.58 ± 0.03		
	tPA	1.58 ± 0.03		
Decreased survival genes:			**Egr‐1	0.42 ± 0.12
			neuro‐D4	0.48 ± 0.01
			trkB	0.48 ± 0.04
			PKCβ	0.38 ± 0.06
			neuronatin α	0.36 ± 0.10
			PKCγ	0.15 ± 0.05
			BDNF	0.09 ± 0.04
			IP3K	0.05 ± 0.01
			ILK	0.05 ± 0.02

	Early versus delayed protective changes			
	1 h		24 h	
Increased survival genes:	**HO‐1**	**2.66 ± 1.16**	**HO‐1**	**2.23 ± 0.30**
	**Egr‐1	8.11 ± 1.80	HSP27	2.60 ± 0.43
	IL‐6	49.6 ± 39.2	HSP10	1.75 ± 0.11
	SOCS‐3	6.76 ± 1.33	JAK2	1.65 ± 0.22
	cFos	4.62 ± 0.31	IGF II BP3	1.59 ± 0.25
	JunB	2.86 ± 0.28	IGF II	1.55 ± 0.03
	cyclin L	2.55 ± 0.30		
	HES‐1	2.07 ± 0.50		
Decreased pathogenic genes:			*NOR‐1	0.30 ± 0.05
			Calcineurin	0.38 ± 0.04
			nNOS	0.17 ± 0.07
			

Note

Categorical filtering was applied to the differentially expressed genes influenced by NMDA exposure. Many of the genes have been primarily linked to either pathogenic events or cell repair/survival. From that list, we tabulated early degenerative changes consisting of up‐regulated pathogenic gene expression, as well as delayed degenerative changes consisting of increased pathogenic genes as well as down‐regulated expression of a putative set of survival genes. Highlighted in bold, MCP and C/EBP exhibited enhancement of expression at early and delayed post‐insult times. Also tabulated are early protective increases in primarily pro‐survival genes, as well as protective down‐regulation of three pathogenic genes. In bold among pro‐survival elements, only HO‐1 exhibited sustained enhancement. Biphasic responding genes are noted with asterisks.

The categorical filtering also found evidence for opposing pathways. Early responses to the NMDA insult consisted of degenerative increases in the 15 primarily pathogenic genes listed above as well as protective increases in eight primarily pro‐survival genes (see Table 2). The 24‐h post‐insult findings were more complicated but still involved opposing pathways. The delayed responses to the excitotoxic episode involved both degenerative changes (up‐regulated pathogenic genes and down‐regulated survival genes) and protective changes (up‐regulation of 6 survival genes and down‐regulation of three pathogenic genes). Among the 14 up‐regulated survival genes, only HO‐1 exhibited sustained enhancement, having increased expression at the early and delayed time points. Regarding the two biphasic responding genes, Egr‐1 expressed an early protective response followed by a delayed degenerative event of down‐regulation. In contrast, the pathogenic gene NOR‐1 expressed an early degenerative response followed by delayed down‐regulation.

The degenerative and protective gene differences were subsequently used to assess networks involved in the distinct pathways. Starting from the gene coexpression networks generated in Figure 3A, all nodes not categorized as pathogenic and/or protective based on literature searches were removed. Next, the remaining nodes were then annotated based on four functional categories (from Table 1): (i) transcription factors, (ii) cytokines, (iii) receptors and channels, and (iv) kinases and transduction, or as ‘other.’ As a result, diagrammed in Figure 3B, gene expression data for pathogenic and protective genes at one and 24 h post‐insult were overlaid onto the coexpression networks to create four networks of differential gene responses. At 1 h post‐insult, both protective and pathogenic genes were up‐regulated and pathogenic gene expression clustering matched that of the coexpression network. In the protective early gene expression network, clustering was less apparent and a wide variety of gene functional categories were observed. At 24 h post‐insult, protective and pathogenic genes were both up‐ and down‐regulated and temporal gene expression data mirrored coexpression clustering.

**FIGURE 4:**
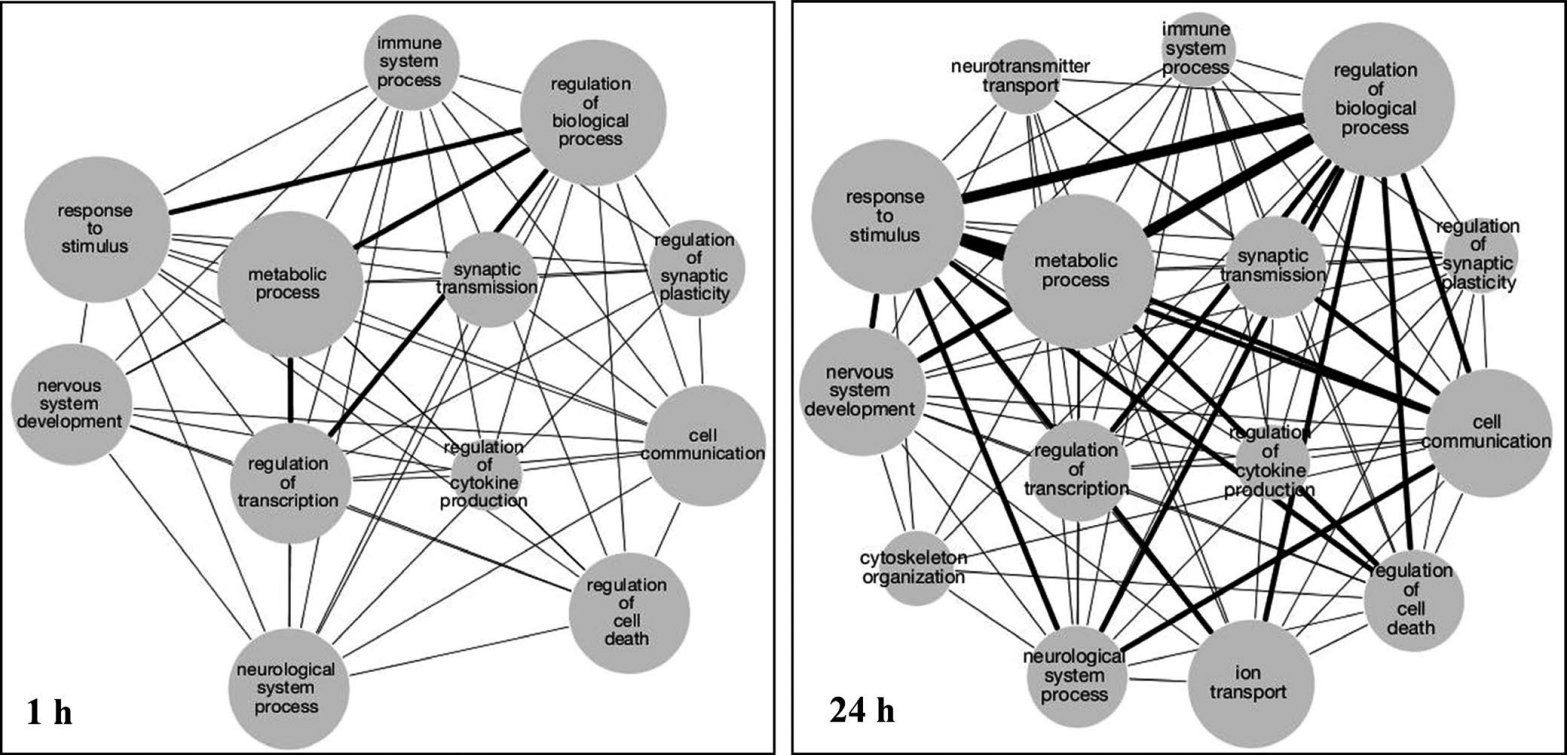
Functional networks of differentially expressed genes after the excitotoxic insult. Gene ontology networks generated from differentially expressed genes 1 (left) and 24 (right) hours after NMDA‐induced excitotoxicity show temporally based functional differences. Network interactions were generated using Cytoscape in which the node size denotes the number of genes with a given functional annotation binned into four groups: 1–10, 11–20, 21–30 and ≥31 genes. Edge size denotes the number of genes shared between the nodes binned into three groups: 1–10, 11–20 and ≥21 genes

To further understand how the temporal changes at a gene expression level might impact system behaviour, functional networks involving the early versus delayed differentially expressed genes were generated by mapping the gene name to biological function using Gene Ontology (GO). Networks were subsequently created using Cytoscape, where each node represents a GO biological function category (Figure 4). Node sizes represent the number of genes within a given GO category while edge sizes denote the number of genes shared between the category nodes.

**FIGURE 5:**
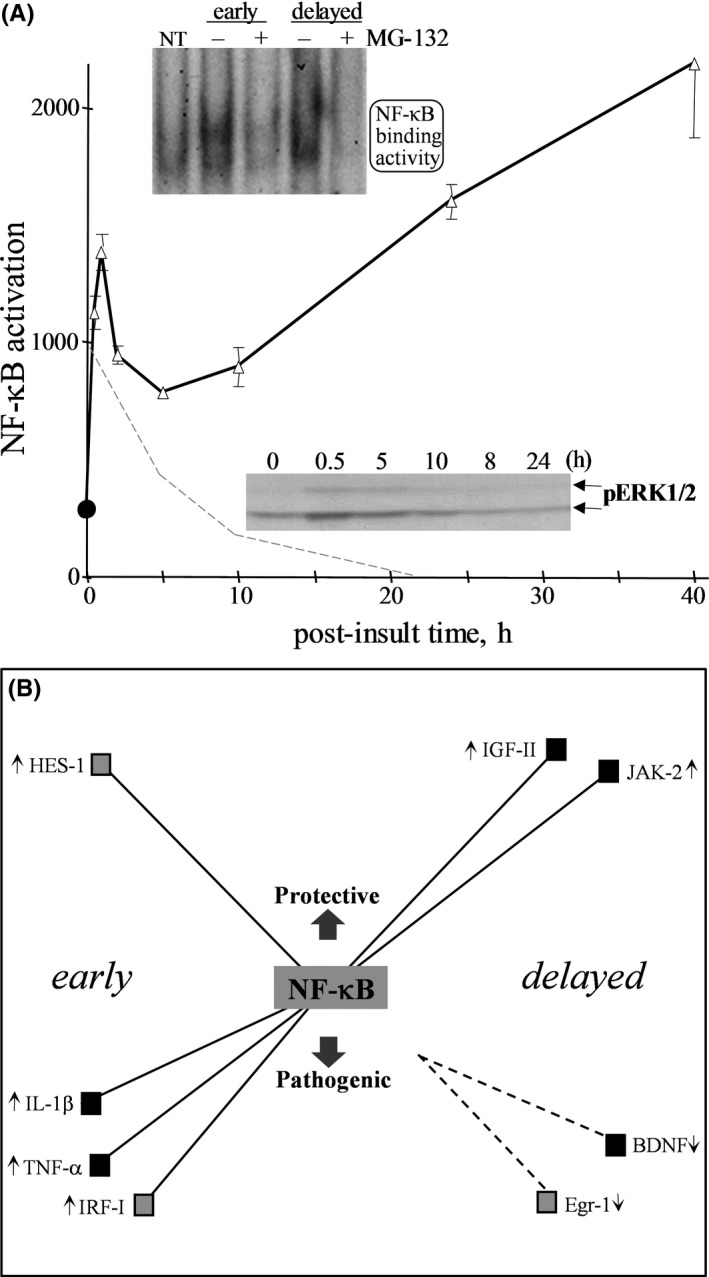
Distinct and biphasic activation of NF‐κB in the excitotoxic hippocampus. NMDA was infused into hippocampal explants for 20 min, followed by rapid washout and antagonist quenching for the defined insult to compare to non‐treated control tissue (NT). Slice cultures were treated with MG‐132 for 1 h prior to the NMDA infusion and during insult and post‐insult periods. Isolated nuclei were assessed for MG‐132‐sensitive NF‐κB activation through probe binding occurring early (1 h) vs. delayed (24 h) after the excitotoxic insult as determined by EMSA (A). The integrated optical densities (means ±SEM) represent NF‐κB‐DNA‐binding activity of control (circle) and NMDA‐treated tissue (triangles), exhibiting the solid line of a biphasic profile (one‐way ANOVA: *p* < 0.0001; *n* = 4–12 across plotted post‐insult times). Single‐phase activation of the ERK1/ERK2 mitogen‐activated protein kinase (MAPK) was found to be an early response in NMDA‐treated explants (lower immunoblot). The phosphorylated active form of ERK2 (pERK2) was assessed for induction over basal levels, resulting in the dashed line profile. Potential opposing roles of NF‐κB in the excitotoxic hippocampus are illustrated in panel B. Following NMDA‐induced excitotoxicity, early and delayed events with connections to NF‐κB are shown for select genes, some of which are transcription factors (grey squares). Evidence also suggests an indirect role NF‐κB may play for the down‐regulation of BDNF and Egr‐1 expression

To identify a candidate signalling avenue activated by NMDA and involved in opposing gene responses, we tested the role of NF‐κB which also served to test the value of the signalling profiles established in the present study. NF‐κB was selected for testing due to being activated by synaptic stimulation,^6^, ^53^ and this transcription factor has been implicated in both cell survival and cell death.^6^, ^54^ A set of 22 opposing genes were first assessed for whether they possess an NF‐κB‐binding site in their promoter region. In the second tier of assessment, the excitotoxic NMDA treatment was applied to hippocampal slices that were also treated with an inhibitor of NF‐κB activity (MG‐132) before, during, and after the NMDA infusion to identify those genes linked to NF‐κB. MG‐132 was confirmed to block the distinct early and delayed phases of NMDA‐induced NF‐κB activation (see Figure 5A). The resulting data listed in Table 3 suggest that NF‐κB is responsible for a subset of opposing genes involved in the brain's response to an excitotoxic episode. The early inductions of IL‐1β, IRF‐I and TNF‐α expression were blocked by the NF‐κB inhibitor, and these pathogenic genes indeed possess a regulatory site for the transcription factor (see rows in bold in Table 3A).

**TABLE 3:** NF‐κB activation blocker MG‐132 identifies opposing genes regulated by NF‐κB in the excitotoxic hippocampus

A) 1 hour post‐insult changes:			C) 24 hours post‐insult changes:		
Pathogenic genes	MG‐132 effect	NF‐κB site	Pathogenic genes	MG‐132 effect	NF‐κB site
Nor‐1 ↑	No	No	MCP ↑	No	Yes
**IL‐1β ↑**	**Yes**	**Yes**	C/EBP ↑	No	No
fra‐1 ↑	No	No	**Caspase 1 ↑**	**Yes**	**Yes**
**IRF‐I ↑**	**Yes**	**Yes**	calpain II ↑	No	No
**TNFα ↑**	**Yes**	**Yes**	cJun ↑	No	Yes

(B)			(D)		
Survival genes	MG‐132 effect	NF‐κB	Survival genes	MG‐132 effect	NF‐κB site
Egr‐1 ↑	No	Yes	HO‐1 ↑	No	No
**HES‐1 ↑**	**Yes**	**Yes**	HSP27 ↑	No	Yes
cFos ↑	No	No	HSP10 ↑	No	Yes
IL‐6 ↑	No	Yes	**JAK2 ↑**	**Yes**	**Yes**
junB ↑	No	No	IGFII BP3 ↑	No	Yes
SOCS‐3 ↑	No	Yes	**IGFII ↑**	**Yes**	**Yes**

Note

Hippocampal explants were treated with 60 μM MG‐132 for 1 h prior to NMDA infusion as well as during the insult and post‐insult incubations. Slices exposed to NMDA alone or in the presence of MG‐132 were harvested one (A, B) or 24 h (C, D) post‐insult. Microarray analyses determined whether MG‐132 blocks the NMDA‐mediated induction of 22 opposing‐acting genes linked to degenerative (A, C) or protective signalling (B, D). Also tabulated is whether the genes possess a regulatory site that binds NF‐κB. Rows in bold denote those with MG‐132 blocking effects.

Besides the degenerative role of NF‐κB regulating the three aforementioned pathogenic genes, NF‐κB also has an apparent regulatory role with an opposing pro‐survival gene, HES‐1, early after the NMDA insult (Table 3B). Note that three other survival genes possess an NF‐κB binding site (Egr‐1, IL‐6 and SOCS‐3), however, the MG‐132 inhibitor had no effect on their NMDA‐induced expression. Of the pathogenic genes induced at 24 h post‐insult, MCP, caspase 1 and cJun have an NF‐κB‐binding site but blocking NF‐κB activity only disrupted the enhanced expression of caspase 1 (Table 3C). Also at 24 h, two NMDA‐induced survival genes with NF‐κB‐binding sites were blocked by the NF‐κB inhibitor (JAK2 and IGFII), whereas three others with binding sites (HSP27, HSP10 and IGFII BP3) did not have their regulated expression blocked by MG‐132 (Table 3D). These data provide an example of determining induced transcriptional responses in order to build an understanding of how a single regulator can influence opposing pathways. Illustrated in Figure 5B are the potential roles NF‐κB has on protective and pathogenic responses in the excitotoxic hippocampus. Note that, based on promoter binding sites, NF‐κB’s regulation of other transcription factors may connect NF‐κB indirectly to the early induction of NOR‐1 (via HES‐1 and/or IRF‐I), Egr‐1 (via HES‐1) and IL‐1β (via IRF‐I) — the IL‐1β induction also has a direct connection to NF‐κB based on the MG‐132 results. Interestingly, results with the MG‐132 inhibitor also suggest indirect effects that may contribute to the delayed down‐regulation of BDNF and Egr‐1, with the latter having the potential to influence NOR‐1 and IP3K as well.

## 4 DISCUSSION

Over‐activation of glutamatergic synapses recapitulates many aspects of brain disorders linked to excitotoxicity. The present study utilized a defined over‐activity period for NMDA‐type glutamatergic receptors and identified early and delayed expression signatures in a vulnerable brain region, the hippocampus. The distinct signatures act together to make up the brain's response to cellular responses that occur early after an excitotoxic insult or in a delayed manner. Informatics methods were combined with the cultured hippocampal tissue model, thereby eliminating systemic variables and allowing the study to focus on the types of cellular pathways activated. In addition, the use focussed gene array analyses resulted in transcriptional responses representing neurobiology‐relevant pathways. The expression profiles appear to consist of competing genetic programmes, and the distinct programmes can be used to evaluate opposing pathways that make up the brain's response to injury.

The pattern of transcriptional responses found early after the excitotoxic stimulation was distinct as compared to delayed responses. Most dramatic changes one hour post‐insult were twofold‐fivefold up‐regulated levels of cytokines and transcription factors involved in inflammatory responses, cell proliferation and apoptosis. Many of the induced signalling components indicate that competing pathways take part in the brain tissue's response to injury.

Adding to the many comprehensive lists of regulated genes from models of excitotoxic insults, this report will help advance understanding of the brain's response to hypoxic/ischaemic episodes that are linked to excitotoxicity. The identified responses to excitotoxic stimulation involved genes with a wide range of functionality. The differentially expressed genes and their classified functional networks vary widely between the early activated gene regulation events and the delayed groups. As a result, distinct patterns of cellular responses were found early after the brief NMDA insult as compared to responses identified 24 hours later. Dramatic changes one hour post‐insult included twofold to fivefold up‐regulated message levels for cytokines and transcription factors involved in inflammation, cell proliferation and apoptosis, suggesting there are competing pathways at the early post‐insult period and/or the presence of different cell types having opposing response signatures. Further, twofold to fourfold changes 24 hours later also exhibited opposing directions and included the down‐regulation of kinases and transduction molecules.

The delayed down‐regulation of cell signalling processes is clearly indicated in the NMDA insult model used, perhaps as part of compensatory pathways for protection/repair that require sufficient time for a cascade of cellular events and crosstalk.^7^, ^8^, ^9^ Gene ontology analysis also showed neurotransmitter transport and other terms appearing only in the delayed networks. As compared to the early changes in biological function nodes, delayed changes exhibited the inclusion of nodes for ion transport, cytoskeleton organization and neurotransmitter transport, thus suggesting repair/compensatory responses and energy‐dependent processes were activated. Interestingly, in addition to the wide range of functional categories among NMDA‐induced up‐ and down‐regulated genes, long non‐coding RNAs (lncRNAs) assessed in a young rat model of lithium/pilocarpine‐induced status epilepticus were found to exhibit up‐ and down‐regulation for wide functional influence.^55^ The modified lncRNAs identified were found to influence the regulation of genes involved in cell proliferation, inflammation, angiogenesis and autophagy.

Findings from the hippocampal explants highlight the brain tissue's complex responses to an excitotoxic insult, which involve opposing pathways and networks. The degree of pathology produced by over‐activated neurons is determined by both the induced degenerative pathways and the induced compensatory signalling as part of repair mechanisms. With the combination of informatics methods and categorical filtering, the current study found evidence for opposing profiles, and the counteracting pathways may provide distinguishable features between different stages of excitotoxic damage. Early changes in gene expression show that opposing pathways occur soon after an excitotoxic insult, in which degenerative activation of pathogenic genes occurs at the same time protective activation ensues regarding opposing survival genes. Of course, opposing pathways may stem from distinct areas of the hippocampal circuitry with distinctly responsive connectivity. While some circuits may respond to intense neuronal activity by initiating degenerative signals, other over‐activated circuits may trigger repair signalling. The present study supports the idea that the brain is not a passive recipient of pathogenic insults and the induction of degenerative processes, but rather, the brain can trigger pathways of cellular repair that counter pathological responses to injury.

Note that opposing proapoptotic and antiapoptotic genes were reported to be induced in the hippocampus of rats subjected to global cerebral ischaemia^56^ as well as in hippocampal explants exposed to a defined NMDA exposure^6^ as used in the present study. Excitotoxicity through the activation of NMDA receptors shares many of the cellular cascades associated with a variety of brain injuries. The utilization of categorical filtering was an additional informatics step used to identify transcriptional regulation events with a high propensity of being involved in cellular degeneration vs. cellular protection. Further analyses in the current study found that the potent transcription factor NF‐κB appears to play a role, at least in part, in both of the pathogenic and protective expression signatures identified in the excitotoxic hippocampus. Such also supports the assertion of improved profile effectiveness by including a categorical informatics step to identify candidate signalling elements, in this case one potentially responsible for opposing gene responses.

NF‐κB has often been linked to neuronal survival, but with many studies also linking it to excitotoxic pathology.^1^ In addition to regulating responses for a wide array of events including cellular proliferation, inflammation and tissue remodelling, NF‐κB signalling facilitates both protection avenues in surviving cells and cell death pathways for the clearance of dying cells. Such a dynamic and dual role is likely important, due to the complexity of cellular decisions, to allow crosstalk between divergent pathways for cells to adapt to stress, repair injuries and foster tissue health. NF‐κB is activated by synaptic over‐stimulation and it represents signalling reported to underlie complex and often opposing responses,^6^, ^54^, ^57^, ^58^ with links to the MAPK pathway during biphasic gene regulation in surviving tissue.^8^ The transcription factor also frequently responds to excitotoxic insults that have an early phase of cytoskeletal and synaptic compromise.^50^, ^51^, ^59^ Interestingly, NMDA exposure in the current study activated pathogenic as well as protective genes in correspondence with putative transcription programmes linked to biphasic NF‐κB signalling. As previously noted,^50^, ^60^ excitotoxic NMDA receptor stimulation is a pathogenic event shared by different neurological disorders with wide ranging aetiologies, including, strokes, ischaemia and brain injuries. In addition, alterations to the receptors may underlie the early degenerative processes in Alzheimer's disease and the memory impairment in ageing. Many of the genes identified that converge on NF‐κB have been associated with the variety of brain disorders, and perhaps the biphasic action of this transcription regulator underlies influential expression signatures stemming from neuronal over‐stimulation. NMDA‐mediated bidirectional regulation of cellular responses may also involve the distinct functions of synaptic vs. extrasynaptic NMDA receptors, an issue being addressed at the level of receptor subunit composition in the two neuronal locations.^61^ Thus, the indication that opposing pathways converge on a single response element may involve (i) different patterns of temporal regulation, (ii) distinct subcellular locals of the activated pathways or (iii) distinct receptor subtypes triggering the transcriptional induction. Our study identifies transcription profiles and functional networks to begin to understand the combinations of the above events that lead to dual influences on cellular survival and death. The usefulness of the informatics data includes constructing putative signalling networks underlying the opposing directions of cellular fate in order to find avenues to offset clinical outcomes.

## CONFLICT OF INTEREST

Dr. Bahr has patents related to neuroprotection and protease regulation (US Patents 8,163,953 and 10,702,571), but they do not involve information in the present study. The remaining authors have no conflicts of interest to declare.

## AUTHOR CONTRIBUTIONS

**Ebru Caba:** Conceptualization (equal); Data curation (equal); Formal analysis (supporting); Investigation (equal); Methodology (equal); Writing‐original draft (equal). **Marcus D. Sherman:** Data curation (equal); Formal analysis (equal); Methodology (equal). **Karen L. G. Farizatto:** Data curation (equal); Formal analysis (equal); Methodology (equal). **Britney Alcira:** Data curation (supporting); Investigation (supporting). **Hsin‐wei Wang:** Data curation (equal); Formal analysis (equal). **Charles Giardina:** Methodology (equal); Supervision (equal). **Dong‐Guk Shin:** Conceptualization (equal); Investigation (equal); Methodology (equal); Supervision (equal). **Conner I. Sandefur:** Data curation (equal); Formal analysis (equal); Methodology (equal); Supervision (equal). **Ben A. Bahr:** Conceptualization (equal); Data curation (equal); Formal analysis (equal); Funding acquisition (equal); Investigation (equal); Methodology (equal); Project administration (equal); Supervision (equal); Validation (equal); Visualization (equal); Writing‐review & editing (equal).

## Data Availability

The data that support the findings of this study are available from the corresponding author upon reasonable request.
